# Longitudinal Associations of High-Fructose Diet with Cardiovascular Events and Potential Risk Factors: Tehran Lipid and Glucose Study

**DOI:** 10.3390/nu9080872

**Published:** 2017-08-21

**Authors:** Zahra Bahadoran, Parvin Mirmiran, Maryam Tohidi, Fereidoun Azizi

**Affiliations:** 1 Nutrition and Endocrine Research Center, Research Institute for Endocrine Sciences, Shahid Beheshti University of Medical Sciences, P.O. Box 19395-4763, Tehran 1985717413, Iran; z.bahadoran@endocrine.ac.ir; 2 Department of Clinical Nutrition and Dietetics, Faculty of Nutrition Sciences and Food Technology, National Nutrition and Food Technology Research Institute, Shahid Beheshti University of Medical Sciences, P.O. Box 19395-4741, Tehran 19816-19573, Iran; 3 Prevention of Metabolic Disorders Research Center, Research Institute for Endocrine Sciences, Shahid Beheshti University of Medical Sciences, P.O. Box 19395-4763, Tehran 1985717413, Iran; tohidi@endocrine.ac.ir; 4 Endocrine Research Center, Research Institute for Endocrine Sciences, Shahid Beheshti University of Medical Sciences, P.O. Box 19395-4763, Tehran 1985717413, Iran; azizi@endocrine.ac.ir

**Keywords:** fructose, cardiovascular diseases, insulin resistance, creatinine, blood pressure

## Abstract

The relationship between fructose and cardiovascular disease (CVD) remains controversial. In this study, we aimed to assess possible association of dietary intakes of fructose with the risk of CVD events in a prospective population-based study. Participants without CVD (*n* = 2369) were recruited from the Tehran Lipid and Glucose Study and followed a mean of 6.7 years. Dietary data were collected using a validated 168 item semi-quantitative food frequency questionnaire. Dietary total fructose (TF) intake was calculated by sum of natural fructose (NF) in fruits and vegetables and added fructose (AF) in commercial foods. Multivariate Cox proportional hazard regression models, adjusted for potential confounders, were used to estimate the risk of CVD across tertiles of dietary fructose. Linear regression models were used to indicate association of fructose intakes with changes of CVD risk factors over the study period. The mean age of participants (43.5% men) was 38.1 ± 13.3 years at baseline. During an average of 6.7 ± 1.4 years of follow-up, 79 participants experienced CVD outcomes. The mean daily intake of TF was 6.4 ± 3.7% of total energy (3.6 ± 2.0 from AF and 2.7 ± 1.8 from NF). Higher consumption of TF (≥7.4% vs. <4.5% of total energy) was accompanied with an increased risk of CVD (HR = 1.81, 95% CI = 1.04–3.15); higher energy intake from AF was also related to incidence of CVD (HR = 1.80, 95% CI = 1.04–3.12), whereas NF was not associated with the risk of CVD outcomes. Both AF and TF were also related to changes of systolic and diastolic blood pressures, waist circumference, serum insulin and creatinine levels, as well as HDL-C. Our data provides further evidence regarding undesirable effects of fructose intake in relation to risk of CVD events.

## 1. Introduction

Fructose, a simple carbohydrate, occurs as free hexoses in fruits and honey, and also in the form of free glucose-fructose mixtures in high-fructose corn syrup (HFCS), or bound together as sucrose [1]. There is an increasing concern regarding the potential role of fructose-containing sweeteners and the development of metabolic and cardiovascular diseases (CVD) [2,3]. A causal link between fructose intake and the global obesity epidemic has also been proposed in recent years [4]. Excess intake of energy from fructose has been shown to be contribute to stimulate hepatic glucose production, de novo lipogenesis, intrahepatic fat accumulation, hyperuricemia, and consequently development of cardiometabolic events [5,6].

Epidemiological studies also revealed that higher consumption of sweetened beverages and fructose may increase the risk of heart disease [7], hypertension and metabolic syndrome [8]. There are some inconsistencies regarding fructose-containing sugars and obesity, cardiometabolic disease and diabetes [9,10]. Although the vast majority of experimental studies hint towards adverse cardiometabolic outcomes following excessive consumption of fructose, the results from some clinical trials do not support a significant detrimental effect of fructose on metabolic health [11,12]. Most epidemiological reports, however, pointed out that intake of fructose-containing sweeteners and beverages are associated with increased body weight and obesity, and interpretation of these results is complicated because a high-fructose diet is shown to be accompanied with a high-calorie and an unhealthy pattern diet [13]. Accordingly, public health policies to eliminate or limit fructose in the diet seems to be premature, and it has been suggested that more epidemiological studies are crucial to clarify current concerns regarding fructose toxicity [13].

To the best of our knowledge, the association of energy intake from fructose in a usual diet is not clear. In this study, we therefore aimed to investigate the possible role of estimated energy intake from total dietary fructose, as well as its natural sources compared to industrial sources, in the development of CVD events, in the framework of a longitudinal population-based study. To clarify potential mediators of this association, we also assessed changes of CVD risk factors during the study follow-up, in relation to intakes of dietary fructose.

## 2. Materials and Methods 

### 2.1. Study Population

The current study used data collected from the Tehran Lipid and Glucose Study (TLGS), an ongoing community-based, prospective study to investigate and prevent non-communicable diseases (NCD). The TLGS, which started in 1987, in a large-scale community-based prospective study on 15,005 individuals aged ≥3 years, a representative sample of residents of district 13 of Tehran, the capital city of Iran [14]. The measurements are repeated every 3 years to assess any change of NCD risk. For the purpose of this study, we recruited adult men and women (aged ≥19 year), with complete data (demographic, anthropometric, biochemical, and dietary), who had participated in the third TLGS examination (2006–2008). Participants were excluded from the final analysis if they reported unusual energy intake (<800 kcal/day or >4200 kcal/day, respectively) (*n* = 170), or were on specific diets for hypertension, diabetes or dyslipidemia (*n* = 409). Participants were also excluded if they had history of CVD at baseline examination (*n* = 90). The remaining participants (*n* = 2382) were followed until March 2014, for a mean period of 6.7 years from the baseline examination. Participants who left the study (*n* = 14) were excluded, and final analyses were conducted on the data of 2369 adults.

### 2.2. Ethical Consideration

Written informed consent was obtained from all participants. The study protocol, based on the ethical guidelines of the 1975 Declaration of Helsinki, was approved by the Ethics Research Council of the Research Institute for Endocrine Sciences, Shahid Beheshti University of Medical Sciences, Tehran, Iran (Code: IR.SBMU.ENDOCRINE.REC. 1395.335).

### 2.3. Demographic, Anthropometric and Lifestyle Measures

Demographic, anthropometric, and biochemical data were assessed at baseline (2006–2008), and again at follow-up examinations. Trained interviewers collected information including demographic data, medical history, medications, and smoking habits, using pretested questionnaires. Weight was measured to the nearest 100 g using digital scales, while the subjects were minimally clothed, without shoes. Height was measured to the nearest 0.5 cm, in a standing position without shoes, using a tape meter. Body mass index was calculated as weight (kg) divided by the square of the height (m^2^). Waist circumference was measured to the nearest 0.1 cm, midway between the lower border of the ribs and the iliac crest at the widest portion, over light clothing, using a soft measuring tape, without any pressure to the body. For measurements of both systolic (SBP) and diastolic blood pressure (DBP), after a 15-min rest in the sitting position, two measurements of blood pressure were taken on the right arm using a standardized mercury sphygmomanometer; the mean of the two measurements was considered to be the blood pressure of the participant.

### 2.4. Biochemical Measures

Over-night fasting blood samples were collected from all study participants, at baseline and again at the follow-up examination. Fasting serum glucose (FSG) was determined by the enzymatic colorimetric method using glucose oxidase. The standard two-hour serum glucose 2-h SG test was performed for all individuals who were prescribed anti-diabetic drugs. Serum creatinine levels were assayed using the kinetic colorimetric Jaffe method. Triglyceride (TG) levels were assessed by enzymatic colorimetric analysis with glycerol phosphate oxidase. High-density lipoprotein cholesterol (HDL-C) was measured after precipitation of the apolipoprotein B containing lipoproteins with phosphotungstic acid. Analyses were performed using Pars Azmoon kits (Pars Azmoon Inc., Tehran, Iran) and a Selectra 2 auto-analyzer (Vital Scientific, Spankeren, The Netherlands). Both inter-assay and intra-assay coefficients of variation of all assays were <5%. In a subsample of the population (*n* = 904), fasting serum insulin (FSI) was measured, by electrochemiluminescence immunoasaay (ECLIA), using Roche Diagnostics kits and the Roche/Hitachi Cobas e-411 analyzer (GmbH, Mannheim, Germany). The intra- and inter-assay coefficients of variation for insulin were 1.2% and 3.5%, respectively.

### 2.5. Assessment of Fructose Intakes

A 168-item food frequency questionnaire (FFQ) was used at the first examination to assess typical food intake over the previous year. Trained dieticians asked participants to designate their intake frequency for each food item consumed during the past year on a daily, weekly, or monthly basis. Portion sizes of consumed foods reported in household measures were then converted to grams. Validity of the FFQ was previously assessed in a random sample, by comparing the data from 2 FFQs, completed 1 year apart; reliability was assessed by comparing the data from the 2 FFQs and 12 patient self-report dietary recalls [15]. Study of reliability, comparative validity, and stability of dietary patterns derived from the FFQ also showed that there was a reasonable reliability and validity of the dietary patterns among the population over time [16].

Energy and nutrient content of foods and beverages were analyzed using the US Department of Agriculture Food Composition Table (FCT) because the Iranian FCT is incomplete, and has limited data on nutrient content of raw foods and beverages [17]. Dietary intakes of added fructose (AF) was defined as fructose intake from industrialized foods and beverages containing beet or cane sugar/molasses, corn sweeteners and invert syrup. Since there are no databases for added sugar content of Iranian food products, the USDA (United States Department of Agriculture) database for added sugar was used to identify the added sugar contents of food items [18]. The most commonly added sugar in Iranian food products is sucrose, while other sweeteners such as corn syrup are not commonly employed. Hence, 50% of added sugar in food products was considered as fructose. Total fructose intake were calculated by summing up natural fructose and added fructose consumed [8].

### 2.6. Definition of Terms 

The CVD risk score was calculated according to the sex-specific “general CVD” algorithms that incorporated age, total cholesterol, HDL-C, systolic blood pressure, treatment for Hypertension (HTN), smoking, and diabetes status [19]. Diabetes was defined as fasting serum glucose ≥126 mg/dL, two-hour serum glucose ≥200 mg/dL or use of anti-diabetic medications [20]. HTN was defined as systolic blood pressure ≥140 mm Hg, diastolic blood pressure ≥90 mm Hg, or current use of antihypertensive medications [21]. Homeostatic model assessment of insulin resistance (HOMA-IR) was defined as follows: fasting insulin (μU/mL) × fasting glucose (mmol/L)/22.5; this index has been developed as a simple, inexpensive, and validated alternative tool for assessment of insulin resistance in epidemiological studies [22,23].

### 2.7. Definition of Outcome 

Details of the collection of cardiovascular outcome data has been described elsewhere [24,25]. A specific outcome for each event was defined according to the international statistical classification of diseases and related health problems, 10th Revision CRITERIA, and the American Heart Association classification for cardiovascular events. CHD-related events comprised cases of definite myocardial infarction (MI) (diagnostic electrocardiogram and biomarkers), probable MI (positive ECG findings, cardiac symptoms or signs, and missing biomarkers; or positive ECG findings and equivocal biomarkers), unstable angina pectoris (new cardiac symptoms or changing symptom patterns and positive ECG findings with normal biomarkers), angiographic-confirmed CHD, and CHD death. Death from CHD or stroke was confirmed by reviewing the death certificate or medical records. Stroke was considered as a new neurological deficit that lasted at least 24 h. Cardiovascular events was defined as any CHD-related event, stroke, or CVD death (definite fatal MI, definite fatal CHD, and definite fatal stroke) [24,26].

### 2.8. Statistical Analysis

The mean (SD) values and the frequency (%) of baseline characteristics of the study population were compared across tertile categories of total fructose intake using analysis of variance or chi-square test, respectively. To investigate the association of dietary fructose intakes with the incidence of CVD events, usual intakes of fructose (g/day) were considered as both continuous (per each 1 SD) and categorical (tertiles) variables in the models.

Time to event was defined as time to end of follow-up (censored cases) or time to having an event, whichever occurred first. We censored participants at the time of death due to non-CVD causes, at the time of leaving the district, or study follow-up end time of March 2014. Cox proportional hazard regression models were used to assess the hazard ratios (HRs) and 95% confidence interval (CI) of fructose intakes for CVD. The potential confounders, included in the models, were CVD risk score, dietary energy intakes, total carbohydrate and fat intake.

Linear associations of baseline dietary fructose intake with changes of serum lipids, blood pressures, and serum creatinine and insulin levels during the follow-up period were estimated using linear regression models with adjustment for age.

All analyses were performed using SPSS version 20 (IBM Corp., Armonk, NY, USA) and STATA version 12 SE (StataCorp LP, TX, USA), with a two-tailed *p*-value < 0.05 being considered significant.

## 3. Results

Mean age of participants (43.5% men) was 38.1 ± 13.3 years, at baseline. During an average of 6.7 ± 1.4 years of follow-up, 79 participants experienced CVD outcomes. Mean daily intake of TF was 6.4 ± 3.7% of total energy (3.6 ± 2.0 from AF and 2.7 ± 1.8 from NF). Mean (SD) contribution of dietary TF to total energy intake was 3.0 ± 1.4%, 6.3 ± 1.9% and 9.7 ± 3.4%, in its first, second and third tertiles, respectively.

The distribution of major known CVD risk factors, biochemical values and dietary factors across tertile categories of TF intakes are provided in Table 1. Compared to the lowest tertile, participants who were in the highest tertile category of TF had higher body mass index, waist circumference, both systolic and diastolic blood pressures, and lower HDL-C levels at baseline (*p* for all <0.05). Serum creatinine levels was also had an increasing trend across increasing TF intakes (89.6, 92.5 and 94.2 μmol/L in the first, second and third tertile, respectively; *p* < 0.05) There was no significant difference between prevalent diabetes (3.4% vs. 5.1%) and HTN (10.3% vs. 8.3%), as well as mean CVD risk score (19.8 vs. 19.9) in the highest compared to the lowest tertile of TF. Dietary intakes of total carbohydrate as well as fiber were increased across increasing consumption of TF (*p* for both <0.05).

The association of dietary fructose intakes and the risk of CVD events (HR and 95% CI) are shown in Table 2. Higher consumption of TF (≥7.4% vs. 4.5% of total energy) was accompanied with an increased risk of CVD (HR = 1.81, 95% CI =1.04–3.15). Similarly, higher energy intake from AF was also related to incidence of CVD (HR= 1.80, 95% CI= 1.04–3.12), whereas NF was not associated to risk of CVD outcomes.

Table 3 shows a linear association of dietary fructose intakes at baseline with changes of CVD risk factors during the follow-up period. A significant positive association was observed between both TF and AF and changes of serum insulin, HOMA-IR, serum creatinine, blood pressures and waist circumference; TF and AF were also negatively associated with HDL-C changes during the follow-up. There was no significant association between NF and CVD risk factors (data not shown).

## 4. Discussion

In this prospective cohort study, conducted on a representative Iranian population, a mean 6.7-year follow-up showed that higher intake of energy from dietary fructose contributed to development of CVD-related events and CVD risk factors; mean energy intake from fructose ≥9.7% increased risk of CVD outcomes by 83%. In the presence of all potentially confounding variables, a 35% and 49% increased risk of CVD was observed per 1-SD increase in the TF and AF intakes, respectively. In contrast to NF, higher intake of both AF and TF was also significantly associated with adverse changes in CVD risk factors. Of note, the magnitude of β coefficient was stronger for AF compared to TF. Significant associations of TF as well as AF with changes in fasting serum insulin levels, HOMA-IR, serum creatinine and blood pressures may be considered as underlying explanations for the association of dietary fructose intakes and development of CVD, in our population.

Although several experimental and short-term clinical investigations provided documents regarding undesirable cardiometabolic effects of fructose and fructose-containing sweeteners, limited and inconsistent data are available regarding plausible long-term cardiometabolic outcomes following consumption of a high-fructose diet. In a large prospective cohort study of women, a significant positive association was reported between regular consumption of sweetened beverages and risk of coronary heart disease (CHD) [7]. Participants in the top quartile of sugar-sweetened beverage had also a 20% higher relative risk of CHD (relative risk = 1.20, 95% CI = 1.09–1.33; *p* for trend < 0.01) [27]. A cross-sectional analysis in the National Health and Nutrition Examination Survey showed that increased fructose intake ≥74 g/day independently associated with higher odds of elevated blood pressure [28]. In our previous study, we showed that dietary fructose intake >12% from energy was related to higher chances of having abdominal, hypertension and metabolic syndrome [8]; in contrast, increased risk of hypertension however was not related to regular consumption of fructose-containing sweeteners in a cross-sectional investigation [11]. Fructose intake was also not associated with the risk for developing hypertension in Nurses’ Health Study 1 (RR = 1.02, 95% CI = 0.99–1.06), Nurses’ Health Study 2 (RR = 1.03, 95% CI = 0.98–1.08), and in Health Professionals Follow-up Study (RR = 0.99, 95% CI = 0.93–1.05) [29]. In our analysis, increasing energy intakes from fructose, especially from industrial sources, tended to be increased both systolic and diastolic blood pressure, as the main mediator of CVD development. Animal studies indicated an upregulation of sodium/chloride transporters and increased blood pressure resulting in a state of salt overload, following a high-fructose diets; other proposed underlying mechanisms of fructose-induced hypertension include activation of vasoconstrictors, inactivation of vasodilators, and over-stimulation of the sympathetic nervous system in exposure to excess fructose intake [30].

Increased serum creatinine, was another potential mediator of CVD events, which we also observed along with increased consumption of fructose. A high serum creatinine concentration is characterized as a marker for increased risk of CVD [31,32]. An enriched-fructose diet found to be related to increased serum creatinine due its adverse effects on both renal tissue and function [33,34].

A main underlying mechanism may explain adverse cardiometabolic outcomes of fructose intake is induction of hepatic lipogenesis and increased circulatory triglycerides levels [35]. A meta-analysis of intervention studies showed that fructose intake <50 g/day had no significant effect on postprandial triglycerides, whereas ≤100 g/day was associated with increased postprandial triglycerides levels [36]. In our study, we did not observe significant association between fructose intake from habitual diet with changes in fasting serum triglycerides during the study period; however, HDL-C levels was inversely dependent to consumption of energy from fructose.

A novel data provided in our study was the association between fructose consumption and long-term changes of serum insulin and insulin resistance index, as the well-known criteria for assessment of insulin/glucose homeostasis. We observed that fructose intake, especially from industrially-food sources, directly increased fasting serum insulin levels as well as HOMA-IR. Although experimental findings confirm fructose-induced impaired insulin signaling [37], the effect of fructose on insulin/glucose metabolism is still a controversial issue [38]; fructose intake within a normal diet enhances glucose metabolism and acts through several mechanisms to facilitate disposal of a dietary carbohydrate load, whereas in an excess amount, fructose may disturb carbohydrate metabolism and β-cell function [39]. The long-term effect of habitual intakes of dietary fructose in epidemiological studies has not been documented.

Inconsistent findings from previous studies regarding metabolic consequences of fructose consumption have been attributed to a confounding effect of calorie intake; several studies failed to observe a direct positive relationship between sugar intake and metabolic disorders when adjusted for total energy intake in their analysis. Instead of absolute intake (g/day), dietary fructose was considered as the percent of total energy intakes in our analysis, an approach could capture potential mediatory effect of energy to development of obesity and metabolic disorders.

Taking into account potential different metabolic effects of fructose derived from different sources (natural or industrial foods), beyond total fructose intake, we separately estimated dietary fructose from both naturally occurring and industrial sources. In agreement with the current beliefs regarding safety of NF occurring in fruits and vegetables, we did not observe a significant association between NF and development of CVD events or changes of cardiometabolic risk factors.

The main strengths of our study was its prospective design, relatively large sample size with long-term follow-up, and detailed data on the well-known CVD risk factors and potential confounders; comprehensive assessment of dietary intakes using a validated comprehensive FFQ also provided us an accurate estimation of dietary fructose intakes. The availability of multiple health examination data allowed us to simultaneously assess different aspects of cardiometabolic risk factors, and also examine the mechanistic pathway of how fructose affect CVD outcomes, which has never been investigated previously.

As inherent in any epidemiological investigation, our study suffered from some limitations. Due to potential changes in an individual’s diet, as well as changes in other cardiometabolic risk factors during the study follow-up, some degree of misclassification might have occurred which could lead to biased estimated hazard ratios. Although the CVD risk score, a validated composed CVD risk factor in our population [40], was adjusted in the Cox models, there may still be residual or unmeasured confounders that could result in biased exposure effect estimates [41].

## 5. Conclusions 

Together, data of the current study support some proposed mechanistic findings regarding cardiometabolic adverse effects of high-fructose diets. These findings reinforce the importance of dietary intervention strategies with an emphasis on reducing intake of fructose-containing sweeteners and replacement of HFCS with other safe sweeteners. Further prospective studies are needed to confirm these associations, including studies with corresponding biomarkers of cardiometabolic diseases, especially pro-inflammatory cytokines, nitric oxide metabolites and oxidative stress surrogates. Clinical trials with longer duration examining dose-response effects of fructose are also needed to assess clinically relevant endpoints.

## Figures and Tables

**Table 1:** Baseline characteristics of the participants across categories of total fructose intakes (*n* = 2369).

Baseline Variables	Total Dietary Fructose *(% of Energy)*		
	Tertile 1 *(<4.5)*	Tertile 2 *(4.5–7.4)*	Tertile 3 *(≥7.4)*
Age (year)	38.3 ± 14.8	37.9 ± 12.4	38.1 ± 12.6
Male (%)	29.6	44.6	55.9 *
Current smokers (%)	6.8	10.8	9.5
Body mass index (kg/m^2^)	26.2 ± 4.7	26.7 ± 4.9	27.0 ± 4.8 *
Waist circumference (cm)	86.2 ± 13.4	88.4 ± 12.9	90.2 ± 13.2 *
Systolic blood pressure (mm Hg)	108 ± 16.1	109 ± 15.3	111 ± 14.6 *
Diastolic blood pressure (mm Hg)	71.4 ± 10.4	72.8 ± 10.3	73.8 ± 10.4 *
Fasting blood glucose (mg/dL)	88.5 ± 16.8	89.0 ± 18.5	89.1 ± 17.1
Serum triglycerides (mg/dL)	129 ± 78.3	135 ± 79.0	136 ± 79.7
HDL-C (mg/dL)	45.1 ± 10.8	42.2 ± 9.8	42.3 ± 10.0 *
Serum creatinine (μmol/L)	89.6 ± 13.4	92.5 ± 14.8	94.2 ± 14.6 *
Diabetes (%)	5.1	3.6	3.4
Hypertension (%)	8.3	8.9	10.3
CVD risk score	19.9 ± 0.8	19.8 ± 0.7	19.8 ± 0.7
Total carbohydrate (% of energy)	55.6 ± 7.6	56.6 ± 6.7	59.4 ± 6.5 *
Total fiber (g/1000 kcal/day)	15.0 ± 6.9	15.9 ± 6.4	17.8 ± 6.6 *
Total fructose (% of energy)	3.0 ± 1.4	6.3 ± 1.9	9.7 ± 3.4 *
*Natural fructose*	0.9 ± 1.3	2.9 ± 0.8	4.3 ± 1.5 *
*Added fructose*	2.0 ± 0.81	3.4 ± 1.3	5.4 ± 2.1 *

Data are mean ± standard deviation (SD) unless stated otherwise (analysis of variance for continuous variables and chi-square test for dichotomous variables was used. * *p* < 0.05.

**Table 2:** The hazard ratio (95% confidence interval) of cardiovascular disease outcomes across tertile categories of dietary fructose.

	*Per SD*	*T2*	*T3*	*p for Trend*
Total fructose		22/790 ^†^	37/790	
*Crude*	1.48 (1.25–1.75)	1.08 (0.59–1.98)	1.83 (1.07–3.16)	0.041
*Adjusted model* ^‡^	1.35 (1.15–1.58)	1.15 (0.62–2.12)	1.81 (1.04–3.15)	0.068
Added fructose		22/790	37/790	
*Crude*	1.60 (1.42–1.80)	1.08 (0.59–1.98)	1.83 (1.07–3.16)	0.041
*Adjusted model* ^‡^	1.49 (1.31–1.69)	1.09 (0.59–2.00)	1.80 (1.04–3.12)	0.058
Natural fructose		24/790	31/790	
*Crude*	1.09 (0.87–1.36)	0.89 (0.55–1.72)	1.27 (0.75–2.18)	0.53
*Adjusted model* ^‡^	1.04 (0.85–1.27)	1.00 (0.56–1.78)	1.19 (0.69–2.05)	0.75

Dietary fructose were entered as % of total energy in the models. Cox regression models were used. ^†^ *n* (number of case)/*N* (total participants). ^‡^ Adjusted for CVD risk score, total energy intakes (kcal/day), dietary intakes of total fats (g/day).

**Table 3:** The association of fructose intakes and changes of cardiovascular risk factors during the study follow-up.

*Cardiovascular Risk*	*Dietary Total Fructose*	*Added Fructose*
Serum insulin	0.117 (0.023, 0.211)	0.230 (0.058, 0.402)
HOMA-IR	0.024 (0.001, 0.048)	0.047 (0.003, 0.092)
Serum creatinine	0.359 (0.211, 0.507)	0.999 (0.736, 1.26)
Triglycerides	0.310 (−0.521, 1.145)	1.060 (−0.437, 2.55)
HDL-C	−0.297 (−0.410, −0.184)	−0.606 (−0.808, −0.404)
Systolic blood pressure	0.217 (0.063, 0.371)	0.640 (0.366, 0.915)
Diastolic blood pressure	0.267 (0.157, 0.376)	0.546 (0.350, 0.741)
Waist circumference	0.387 (0.252, 0.522)	0.857 (0.617, 1.09)

Data are β regression (95% confidence interval). Age-adjusted linear regression models were used.
